# Supplementary material for: On the origin of low-valent uranium oxidation state
Source: Nat Commun. 2024 Aug 10;15:6861. doi: 10.1038/s41467-024-50924-7 (PMC11316815; doi:10.1038/s41467-024-50924-7)
Supplement: Supplementary file 2 — Supplementary Information [file 41467_2024_50924_MOESM2_ESM.pdf]

# Supplementary information for "On the origin of low-valent uranium oxidation state"

C. L. Silva,[1,2] L. Amidani,[1,2] M. Retegan,[3] E. F. Bazarkina,[1,2] S. Weiss,[2] T. Graubner,[4] F. Kraus,[4] and K. O. Kvashnina[1,2*]

[1] *The Rossendorf Beamline at ESRF – The European Synchrotron, CS40220, 38043 Grenoble Cedex, France*
[2] *Helmholtz-Zentrum Dresden-Rossendorf (HZDR), Institute of Resource Ecology, 01314 Dresden, Germany*
[3] *European Synchrotron Radiation Facility (ESRF), CS40220, 38043 Grenoble Cedex, France*
[4]*Fachbereich Chemie, Philipps-Universität Marburg, Hans-Meerwein-Str. 4, 35032 Marburg, Germany*

## A. Supplementary tables and figures

Supplementary Table 1. ***Ab initio* Hartree−Fock values used for the initial, intermediate, and final electronic configurations of $U^{III}$ and $U^{IV}$**

| | $U^{III}$ | | | $U^{IV}$ | | |
|---|---|---|---|---|---|---|
| **Parameters** | **$5f^3$** | **$3d^95f^4$** | **$4f^{13}5f^3$** | **$5f^2$** | **$3d^95f^3$** | **$4f^{13}5f^2$** |
| $F^2$(5f,5f) | 8.86 | 9.43 | 9.37 | 9.51 | 10.02 | 9.96 |
| $F^4$(5f,5f) | 5.75 | 6.14 | 6.10 | 6.22 | 6.57 | 6.53 |
| $F^6$(5f,5f) | 4.20 | 4.50 | 4.47 | 4.57 | 4.83 | 4.80 |
| ζ(5f) | 0.24 | 0.28 | 0.27 | 0.26 | 0.26 | 0.26 |
| $F^2$(3d,5f) | | 2.36 | | | 2.56 | |
| $F^4$(3d,5f) | | 1.09 | | | 1.19 | |
| $G^1$(3d,5f) | | 1.83 | | | 2.00 | |
| $G^3$(3d,5f) | | 1.11 | | | 1.21 | |
| $G^5$(3d,5f) | | 0.77 | | | 0.85 | |
| ζ(3d) | | 73.39 | | | 73.38 | |
| $F^2$(4f,5f) | | | 4.84 | | | 5.21 |
| $F^4$(4f,5f) | | | 2.07 | | | 2.26 |
| $F^6$(4f,5f) | | | 1.28 | | | 1.39 |
| $G^0$(4f,5f) | | | 1.27 | | | 1.38 |
| $G^2$(4f,5f) | | | 1.58 | | | 1.72 |
| $G^4$(4f,5f) | | | 1.23 | | | 1.34 |
| $G^6$(4f,5f) | | | 0.96 | | | 1.05 |
| ζ(4f) | | | 3.08 | | | 3.08 |

Supplementary Table 2. **Slater-Condon integrals and spin-orbit coupling constants for initial, intermediate, and final electronic configuration of $U^{III}$.** The reduction factors used in the investigation of the different contributions of the RIXS process (Fig. 2) are also shown.

| **Parameters** | **Hartree-Fock values (eV)** | | | **Scaling of Slater-Condon parameters** | | | | | | | | |
|---|---|---|---|---|---|---|---|---|---|---|---|---|
| | | | | **Figure 2a** | | | **Figure 2b** | | | **Figure 2c** | | |
| | **$5f^3$** | **$3d^9 5f^4$** | **$4f^{13} 5f^3$** | **GS** | **IS** | **FS** | **GS** | **IS** | **FS** | **GS** | **IS** | **FS** |
| $F^2(5f,5f)$ | 8.86 | 9.43 | 9.37 | 0.8 | 0.8 | | 0.8 | 0 | | 0.8 | 0.8 | |
| $F^4(5f,5f)$ | 5.75 | 6.14 | 6.10 | 0.8 | 0.8 | | 0.8 | 0 | | 0.8 | 0.8 | |
| $F^6(5f,5f)$ | 4.20 | 4.50 | 4.47 | 0.8 | 0.8 | | 0.8 | 0 | | 0.8 | 0.8 | |
| $\zeta(5f)$ | 0.24 | 0.28 | 0.27 | 1.0 | 1.0 | | 1.0 | 1.0 | | 1.0 | 1.0 | |
| $F^2(3d,5f)$ | | 2.36 | | | 0.8 | | | 0 | | | 0.8 | |
| $F^4(3d,5f)$ | | 1.09 | | | 0.8 | | | 0 | | | 0.8 | |
| $G^1(3d,5f)$ | | 1.83 | | | 0.8 | | | 0 | | | 0.8 | |
| $G^3(3d,5f)$ | | 1.11 | | | 0.8 | | | 0 | | | 0.8 | |
| $G^5(3d,5f)$ | | 0.77 | | | 0.8 | | | 0 | | | 0.8 | |
| $\zeta(3d)$ | | 73.39 | | | 1.0 | | | 1.0 | | | 1.0 | |
| $F^2(4f,5f)$ | | | 4.84 | | | 0.8 | | | 0.8 | | | 0 |
| $F^4(4f,5f)$ | | | 2.07 | | | 0.8 | | | 0.8 | | | 0 |
| $F^6(4f,5f)$ | | | 1.28 | | | 0.8 | | | 0.8 | | | 0 |
| $G^0(4f,5f)$ | | | 1.27 | | | 0.8 | | | 0.8 | | | 0 |
| $G^2(4f,5f)$ | | | 1.58 | | | 0.8 | | | 0.8 | | | 0 |
| $G^4(4f,5f)$ | | | 1.23 | | | 0.8 | | | 0.8 | | | 0 |
| $G^6(4f,5f)$ | | | 0.96 | | | 0.8 | | | 0.8 | | | 0 |
| $\zeta(4f)$ | | | 3.08 | | | 1.0 | | | 1.0 | | | 1.0 |

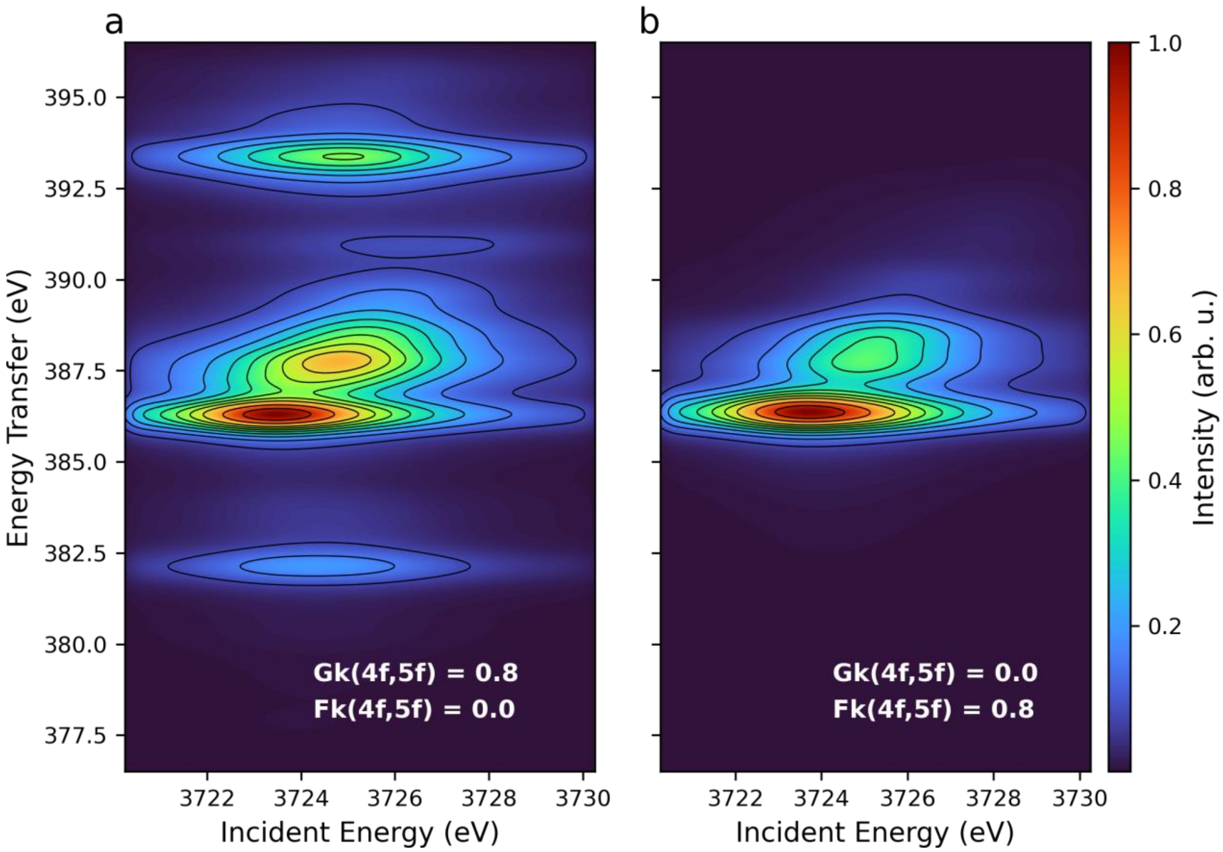


Supplementary Fig 1. **Role of the exchange ($G^k$) and electrostatic ($F^k$) interactions in the RIXS maps at $U^{III}$ $M_4$ edge. a** Fk(4f,5f) parameter is negligible. **b** Gk(4f,5f) is negligible. For both calculations, the reduction factor of the Slater integrals for the initial and intermediate states was kept at 0.8. The two features seen below and above the main RIXS intensity maximum (Fig. 2a) are due to the interaction of the 4f core hole with the 5f electrons in the final state of the spectroscopic process, and they are dependent on the values of exchange integrals Gk(4f,5f).

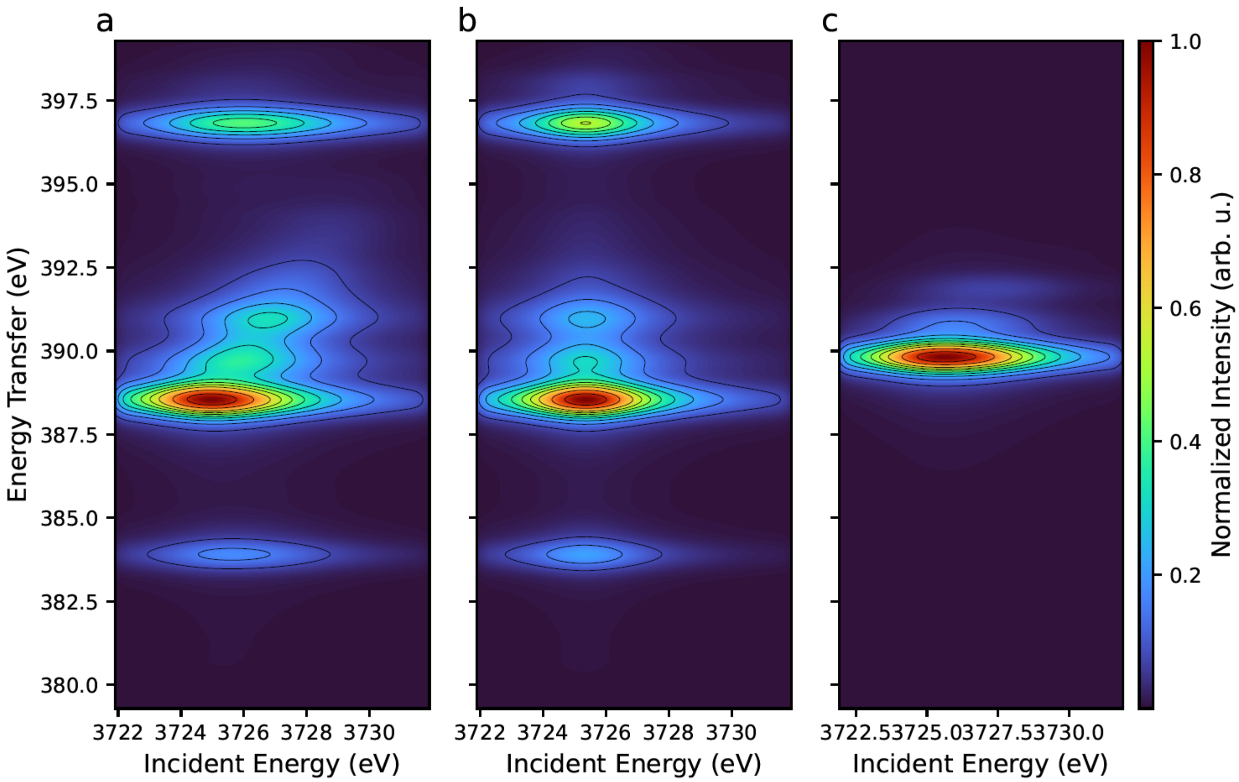


Supplementary Fig 2. **Calculated RIXS at $U^{IV}$ $M_4$ edge for $5f^2$ ground state configuration. a** All Slater-Condon integrals are considered and reduced to 80 % of their atomic value. **b** Intermediate-state electron-electron interactions are neglected. **c** Final-state electron-electron interactions are neglected.

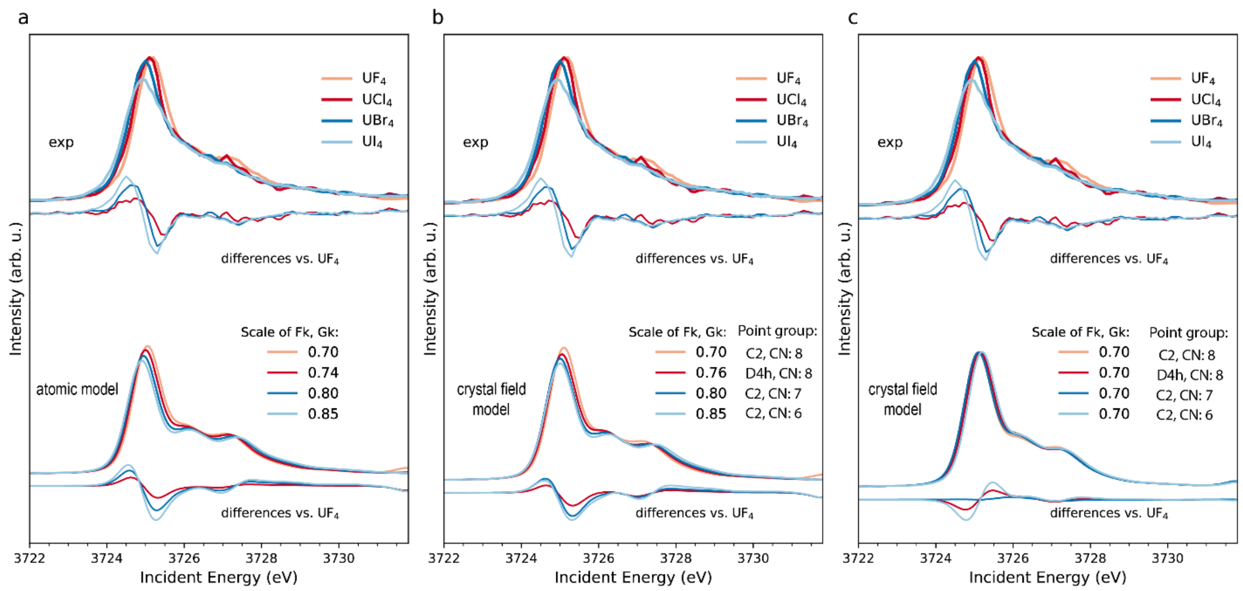


Supplementary Fig 3. **Investigation of the optimal model to reproduce the HERFD-XANES data at U $M_4$-edge for the $U^{IV}$ halide systems.** The top panel shows the experimental data compared to the calculations of each model at the bottom. **a** Atomic model with a 15 % reduction in Slater-Condon integrals from $UI_4$ to $UF_4$. **b** Combination of the crystal field model including the local geometry and the modulation of the Slater-Condon integrals. **c** Crystal field model without modulation of the Slater-Condon integrals. To quantitatively compare the results, $UF_4$ is taken as the reference, and the spectral differences between the series are plotted at the bottom. The differences indicate that the changes in panels (a) and (b) are in the direction of the experimental changes. This analysis shows that the intra-atomic interactions are the principal force shaping the U $M_4$ edge data for these materials.

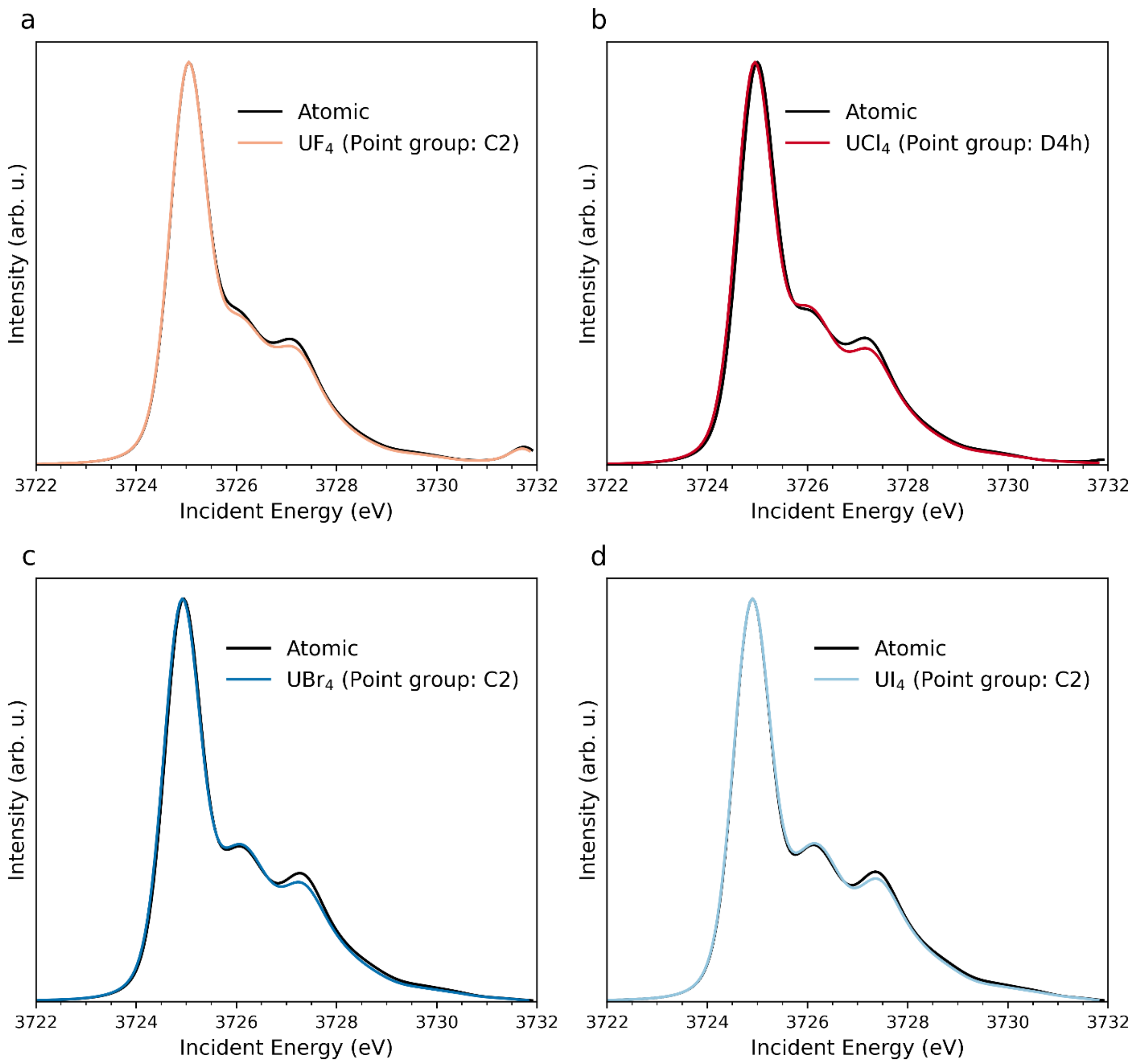


Supplementary Fig 4. **Comparison between atomic and crystal field HERFD-XANES calculations at the $U^{IV}$ $M_4$-edge** for **a** $UF_4$, **b** $UCl_4$, **c** $UBr_4$, **d** $UI_4$. Compared to purely atomic calculations, the crystal field slightly reduces the second post-edge feature.

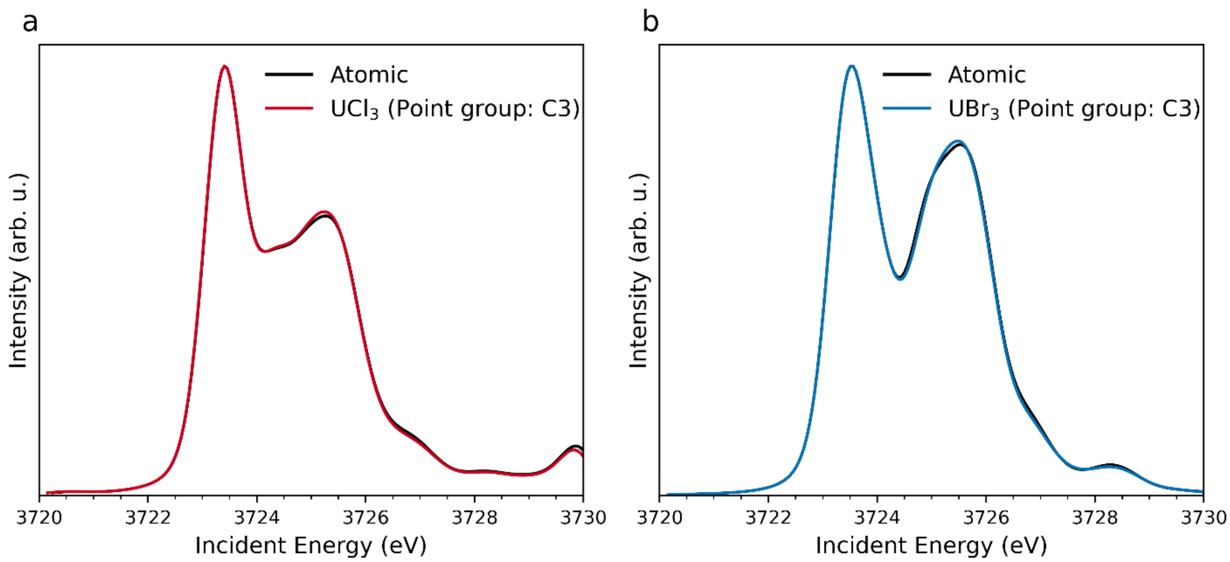


Supplementary Fig 5. **Comparison between atomic and crystal field HERFD-XANES calculations at the $U^{III}$ $M_4$-edge** for **a** $UCl_3$ and **b** $UBr_3$.

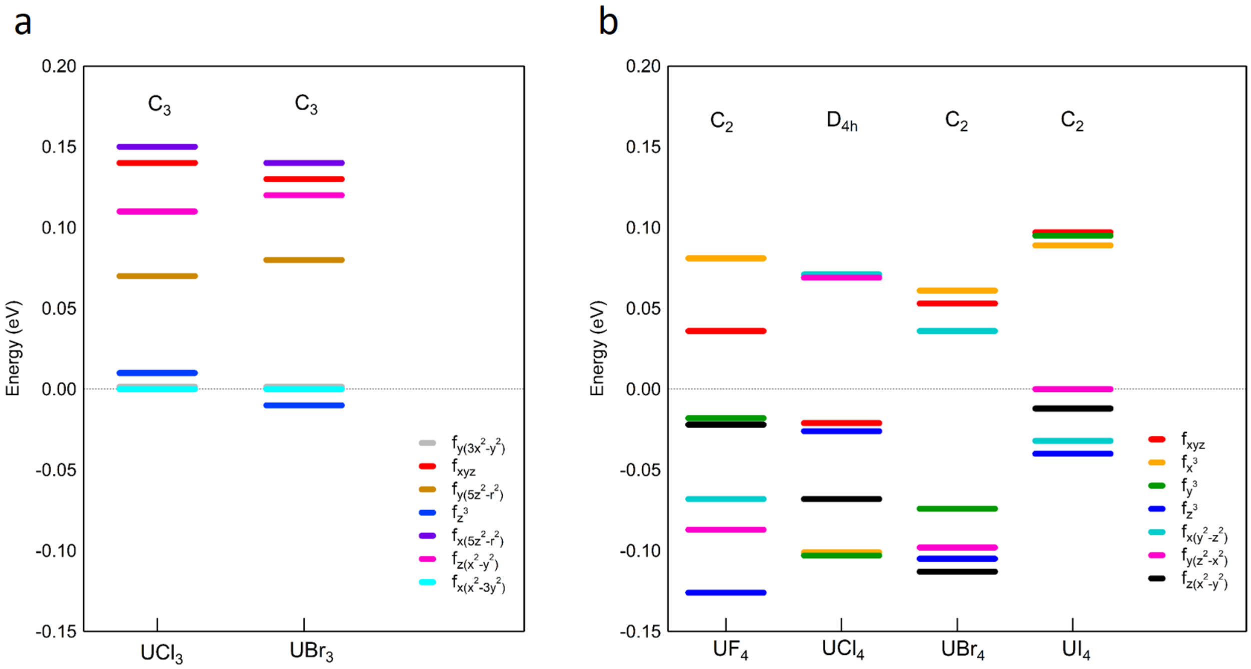


Supplementary Fig 6. **Splitting of the 5f orbitals due to the crystal field for (a)** $UX_3$ and **(b)** $UX_4$ systems.

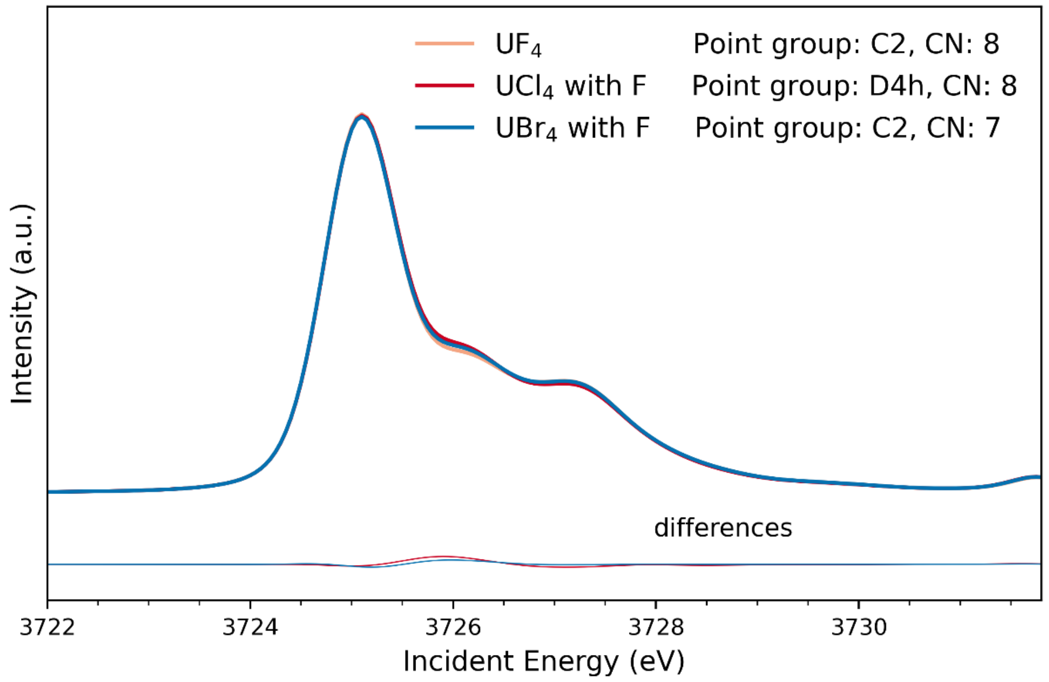


Supplementary Fig 7. **Investigation of the effects of the coordination number (CN) and local geometry around the absorbing atom.** The calculations were performed for $UCl_4$ and $UBr_4$ with Cl and Br replaced by F.

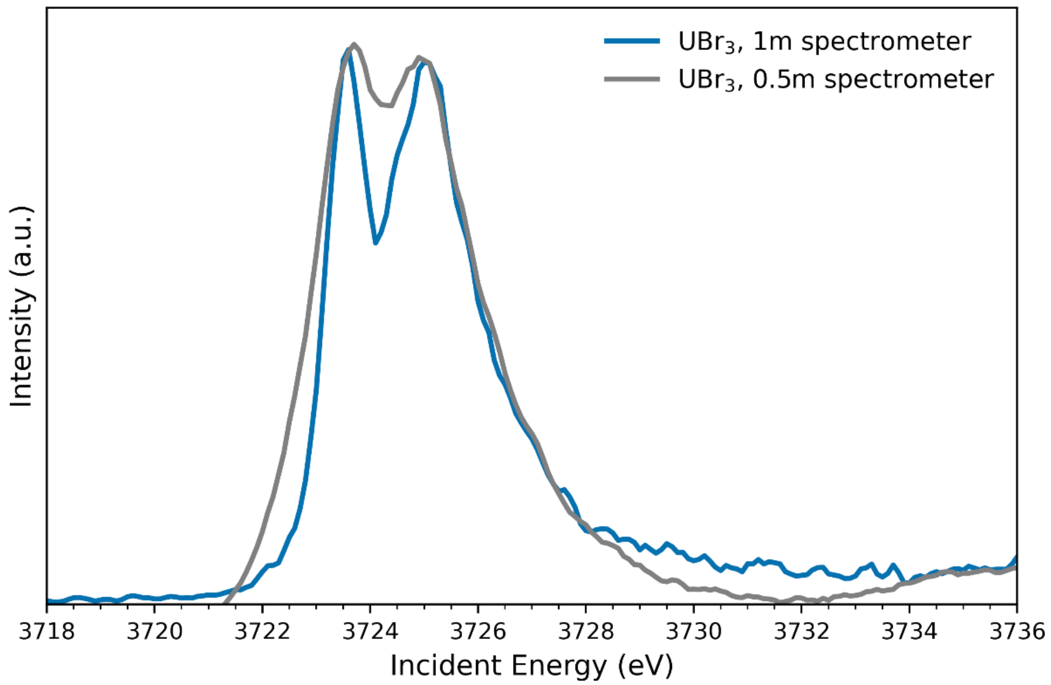


Supplementary Fig 8. **Beam damage analysis.** HERFD-XANES experimental data for $UBr_3$ in different configurations of the spectrometer.

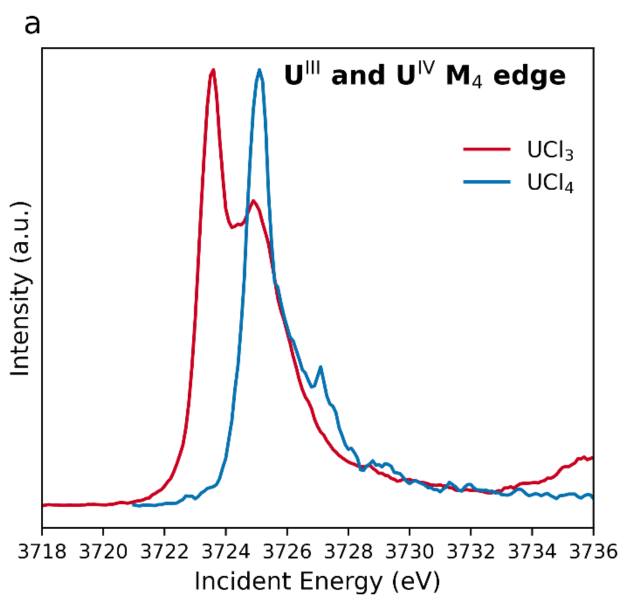


Supplementary Fig 9. **HERFD XANES data for the ground state configuration of $5f^3$ and $5f^2$ for different actinide compounds. a** U systems. **b** Pu systems. $U^{III}$ and $Pu^{III}$ species show a double peak shape.

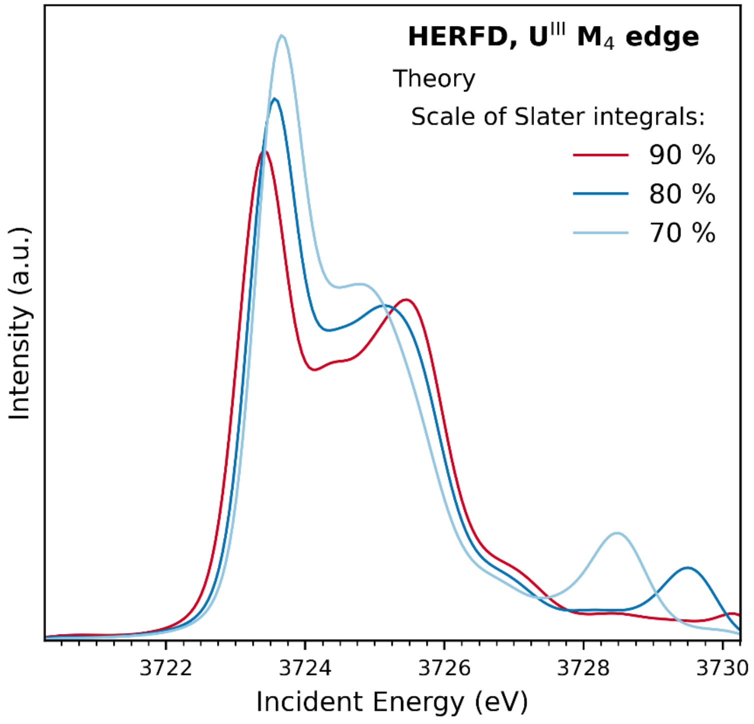


Supplementary Fig 10. **Atomic HERFD-XANES calculations at $U^{III}$ $M_4$ edge when the reduction factor of Slater-Condon integrals is varied by the same amount for the ground-state, intermediate-state, and final-state of the RIXS process.**

a

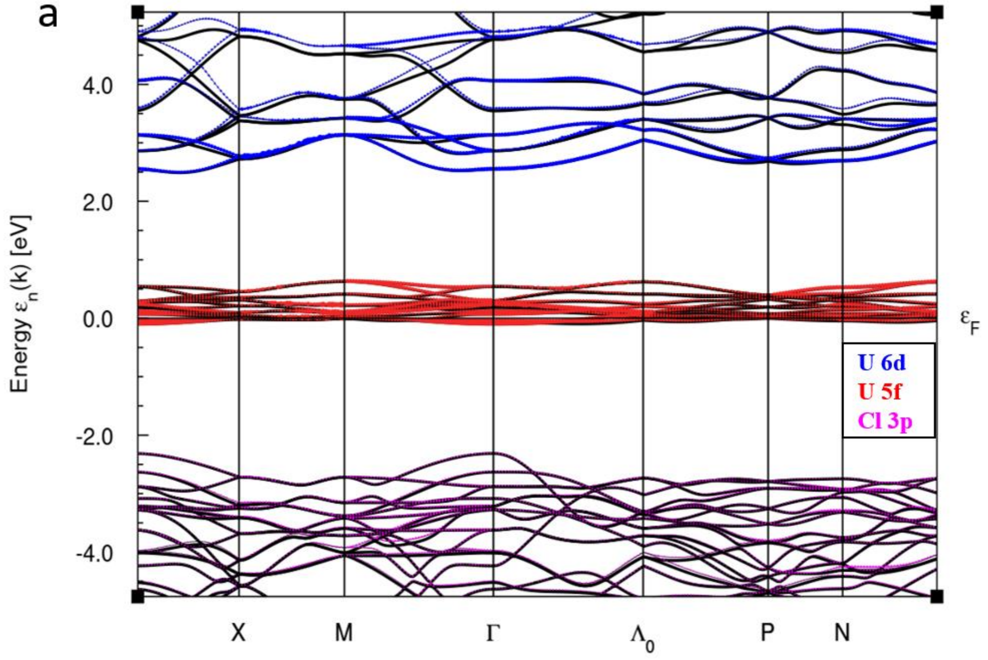


b

| | $f_{xyz}$ | $f_{x(5x^2-r^2)}$ | $f_{y(5y^2-r^2)}$ | $f_{z(5z^2-r^2)}$ | $f_{x(y^2-z^2)}$ | $f_{y(z^2-x^2)}$ | $f_{z(x^2-y^2)}$ |
|---|---|---|---|---|---|---|---|
| $f_{xyz}$ | -0.022 | 0. | 0. | 0. | 0. | 0. | 0. |
| $f_{x(5x^2-r^2)}$ | 0. | -0.103 | 0. | 0. | 0.114 | 0. | 0. |
| $f_{y(5y^2-r^2)}$ | 0. | 0. | -0.103 | 0. | 0. | -0.114 | 0. |
| $f_{z(5z^2-r^2)}$ | 0. | 0. | 0. | -0.026 | 0. | 0. | 0. |
| $f_{x(y^2-z^2)}$ | 0. | 0.114 | 0. | 0. | 0.069 | 0. | 0. |
| $f_{y(z^2-x^2)}$ | 0. | 0. | -0.114 | 0. | 0. | 0.069 | 0. |
| $f_{z(x^2-y^2)}$ | 0. | 0. | 0. | 0. | 0. | 0. | -0.068 |

Supplementary Fig 11. **Wannierization process for $UCl_4$. (a)** Electronic bandstructure calculations. The background (in black) is the electronic band structure calculated by FPLO. The projected Wannier bands are shown in different colours according to each orbital (blue for U 6d, red for U 5f, pink for Cl 3p). **(b)** Numerical crystal field Hamiltonian matrix obtained following wannierization. The eigenstates (diagonal elements) and mixing parameters (off-diagonal elements) values in cubic harmonics, using a basis of symmetric functions for the D4h point group, were applied to calculate the 3d4f RIXS process using a crystal field multiplet model.

a

## $UF_4$

| | | | | | | |
|---|---|---|---|---|---|---|
| 0.052 | 0. | 0. | -0.036 | 0. | 0. | 0.026 |
| 0. | 0.059 | -0.031 | 0. | -0.005 | 0.015 | 0. |
| 0. | -0.031 | -0.074 | 0. | 0.001 | 0.031 | 0. |
| -0.036 | 0. | 0. | -0.105 | 0. | 0. | -0.041 |
| 0. | -0.005 | 0.001 | 0. | 0.034 | 0.027 | 0. |
| 0. | 0.015 | 0.031 | 0. | 0.027 | -0.098 | 0. |
| 0.026 | 0. | 0. | -0.041 | 0. | 0. | -0.113 |

b

## $UBr_4$

| | | | | | | |
|---|---|---|---|---|---|---|
| 0.035 | 0. | 0. | -0.146 | 0. | 0. | 0.006 |
| 0. | 0.079 | -0.092 | 0. | 0.036 | 0.01 | 0. |
| 0. | -0.092 | -0.018 | 0. | -0.046 | -0.053 | 0. |
| -0.146 | 0. | 0. | -0.126 | 0. | 0. | 0.019 |
| 0. | 0.036 | -0.046 | 0. | -0.07 | -0.072 | 0. |
| 0. | 0.01 | -0.053 | 0. | -0.072 | -0.087 | 0. |
| 0.006 | 0. | 0. | 0.019 | 0. | 0. | -0.022 |

c

## $UI_4$

| | | | | | | |
|---|---|---|---|---|---|---|
| 0.096 | 0. | 0. | 0.121 | 0. | 0. | 0.032 |
| 0. | 0.087 | 0.044 | 0. | 0.052 | -0.018 | 0. |
| 0. | 0.044 | 0.095 | 0. | 0.03 | 0.029 | 0. |
| 0.121 | 0. | 0. | -0.04 | 0. | 0. | -0.013 |
| 0. | 0.052 | 0.03 | 0. | -0.034 | 0.099 | 0. |
| 0. | -0.018 | 0.029 | 0. | 0.099 | 0. | 0. |
| 0.032 | 0. | 0. | -0.013 | 0. | 0. | -0.012 |

Supplementary Fig 12. **Crystal field Hamiltonian matrices obtained after wannierization for (a)** $UF_4$, **(b)** $UBr_4$, and **(c)** $UI_4$. The eigenstates (diagonal elements) and mixing parameters (off-diagonal elements) values in cubic harmonics, using a basis of symmetric functions for the C2 point group, were applied to calculate the 3d4f RIXS process using a crystal field multiplet model.

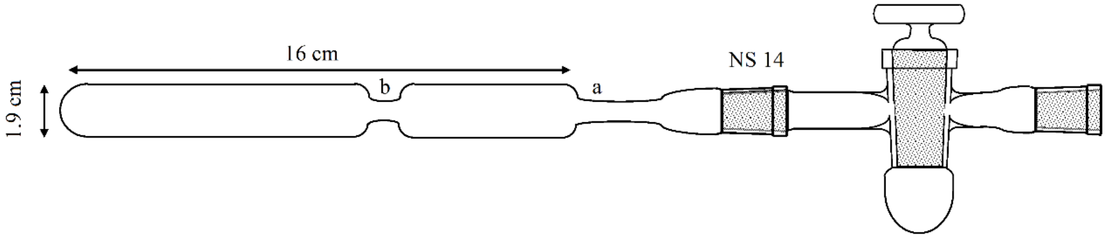


Supplementary Fig 13. **Ampoule used for the synthesis and purification of uranium(IV) halides.** These ampoules are equipped with a NS14.5 ground joint and a constriction at position a for eased flame sealing. A constriction at position b at one third of the ampoule length is used for facilitated breakup of the ampoule and prevents mixing of loose crystals in the sink with residual powder in the source of the transport ampoule. The length of the sealed ampoule is 16 cm, the outer diameter is 19 mm with 1.3 mm wall thickness, resulting in approximately 34 mL of volume. The pressure resistance according to Schott Duranglas GmbH is 10.3 ± 0.8 bar. The ampoules are filled inside a glove box with a long funnel to prevent contaminating the sink and the sealing constraint with nonvolatile substances, which is problematic for the flame sealing. A stopcock attached to the ground joint allows the transfer to a Schlenk line. There the content of the ampoule is cooled with liquid nitrogen and the ampoule is flame sealed under vacuum after pumping off the argon. Batch size is limited due to diffusion problems. Batches, larger than ~1 g of $UO_2$ often lead to reduced yield due to a clogged ampoule.

## B. Supplementary Methods

### Quantum Theory of Atoms in Molecules (QTAIM) calculations

The electron densities required for the QTAIM analysis were calculated using the ORCA software package.[1] In the calculations, we used structural models that included the uranium atom and the bound halogen ligands. As in the case of the FPLO calculations, the PBE density functional was used together with the contracted version def2-TZVP basis set for F, Cl, Br, and I[2,3] and the def2-TZVPP basis set for U.[4] The resolution of identity approximation and appropriate basis set were used to speed up the calculation.[5] The scalar relativistic effects were accounted for using the Douglas−Kroll−Hess Hamiltonian.[6] The missing surrounding atoms were included using the CPCM model.[7] A typical ORCA input file is listed below.

```
! uks pbe dkh dkh-def2-tzvp sarc/j cpcm
! printbasis uno keepdens notrah kdiis soscf

% basis
    newgto U "SARC-DKH-TZVPP" end
    end

% pal
    nprocs 10
    end

* xyz -4 3
U             1.24864500    -2.28545000    -1.69777500
F            -0.82273600    -1.62217500    -1.10898700
F             1.36955400    -3.19597500    -3.86481500
F             0.72261700    -0.56437500    -3.08383900
F            -0.10195200    -4.10865000    -1.92459800
F             1.77467300    -0.56437500    -0.31171100
F             2.59924200    -4.10865000    -1.47095200
F             3.32002600    -1.62217500    -2.28656300
F             1.12773600    -3.19597500     0.46926500
*
```

The electron density was written to a cube file using a dense grid of 80 points along each axis. The program Critic2[8] was then used to determine the QTAIM parameters. It should be noted that the program implements an accurate algorithm to determine the critical points sampled using grids.[9] A typical Critic2 input file is listed below.

```
molecule input.eldens.cube

load input.eldens.cube smoothrho

auto discard "$1 < 1e-5"

cpreport input.cif

cpreport input.vmd graph
```

## REFERENCES


1. Neese, F. Software update: The ORCA program system—Version 5.0. *WIREs Comput. Mol. Sci.* **12**, e1606 (2022).
2. Weigend, F. & Ahlrichs, R. Balanced basis sets of split valence, triple zeta valence and quadruple zeta valence quality for H to Rn: Design and assessment of accuracy. *Phys. Chem. Chem. Phys.* **7**, 3297 (2005).
3. Rolfes, J. D., Neese, F. & Pantazis, D. A. All-electron scalar relativistic basis sets for the elements Rb–Xe. *J Comput Chem* **41**, 1842–1849 (2020).
4. Pantazis, D. A. & Neese, F. All-Electron Scalar Relativistic Basis Sets for the Actinides. *J. Chem. Theory Comput.* **7**, 677–684 (2011).
5. Weigend, F. Accurate Coulomb-fitting basis sets for H to Rn. *Phys. Chem. Chem. Phys.* **8**, 1057 (2006).
6. Heß, B. A., Marian, C. M., Wahlgren, U. & Gropen, O. A mean-field spin-orbit method applicable to correlated wavefunctions. *Chem. Phys. Lett.* **251**, 365–371 (1996).
7. Garcia-Ratés, M. & Neese, F. Effect of the Solute Cavity on the Solvation Energy and its Derivatives within the Framework of the Gaussian Charge Scheme. *J. Comput. Chem.* **41**, 922–939 (2020).
8. Otero-de-la-Roza, A., Johnson, E. R. & Luaña, V. Critic2: A program for real-space analysis of quantum chemical interactions in solids. *Comput. Phys. Commun.* **185**, 1007–1018 (2014).
9. Otero-de-la-Roza, A. Finding critical points and reconstruction of electron densities on grids. *J. Chem. Phys.* **156**, 224116 (2022).
